# Can Artificial Intelligence Software be Utilised for Thyroid Multi‐Disciplinary Team Outcomes?

**DOI:** 10.1111/coa.14305

**Published:** 2025-03-20

**Authors:** Amir Habeeb, Kim Hui Lim, Xenofon Kochilas, Nazir Bhat, Furrat Amen, Samuel Chan

**Affiliations:** ^1^ Academic Clinical Fellow Association Queen Mary University of London London UK; ^2^ Ear, Nose and Throat Surgery Department Peterborough City Hospital Peterborough UK

**Keywords:** artificial intelligence, head and neck, malignancy, multidisciplinary team, otorhinolaryngology, outcomes, thyroid

## Abstract

**Objectives:**

ChatGPT is one of the most publicly available artificial intelligence (AI) softwares. Ear, nose and throat (ENT) services are often stretched due to the increasing incidence of thyroid malignancies. This study aims to investigate whether there is a role for AI software in providing accurate thyroid multidisciplinary team (MDT) outcomes.

**Methods:**

A retrospective study looking at unique thyroid MDT outcomes between October 2023 and May 2024. ChatGPT‐4TM was used to generate outcomes based on the British Thyroid Association (BTA) Guidelines for Management of Thyroid Cancer. Concordance levels were collected and analysed.

**Results:**

Thirty thyroid cases with a mean age of 58 (*n* = 24 female, *n* = 6 male) were discussed. The MDT's outcome had a 100% concordance with BTA Guidelines. When comparing ChatGPT‐4TM and our MDT the highest level of concordance Y1 was seen in 67% of case while 13% of cases were completely discordant.

**Conclusions/Significance:**

AI is cheap, easy to use can optimise complex thyroid MDT decision making. This could free some clinicians allowing them to meet other demands of the ENT service. Some key issues are the inability to completely rely on the AI software for outcomes without being counterchecked by a clinician.

**Summary**

- Artificial intelligence is a growing field with endless applications in clinical medicine, from diagnostics to therapeutics.
- Thyroid cancer is increasing in incidence, and accompanying this is more pressure on the ear, nose and throat surgery service.
- This study was able to show a 67% highest level of concordance with clinician‐led thyroid cancer multi‐disciplinary team decisions and only 13% were completely discordant. The remaining 20% were acceptable outcomes but not the gold standard according to British Thyroid Association guidelines.
- Artificial intelligence software such as ChatGPT‐4 may be effective in optimising complex thyroid multi‐disciplinary team decisions, which could free up clinicians for other parts of the service.
- There is still a need at present for counterchecking decisions made by artificial intelligence software by a clinician.

## 1 Introduction

Artificial intelligence (AI) is an increasing topic of debate in recent years, with the promise of improved efficiency and productivity in several industries. The Healthcare industry remains no exception in the desire to benefit from this emerging technology; however, there is reluctance globally to implement this clinically [1, 2].

AI technologies in healthcare have been applied to a number of different facets, including diagnostics, therapeutics, population health management as well as administration and regulation of a health service. In therapeutics, AI has shown effectiveness in extracting clinical guidelines and applying them to electronic patient records, providing holistic and evidence‐based treatment options [2, 3]. In the context of thyroid pathology, AI has been applied to areas of diagnostic and therapeutic decision making such as cytology, histopathology and thyroid imaging. For example, in cytology AI models have been able to show an enhanced accuracy of fine‐needle aspiration analysis due to more sophisticated methods of identifying malignant cellular patterns [4, 5, 6]. This would subsequently reduce the large inter‐observer variability seen in thyroid cytology classification and consequently impact multi‐disciplinary team (MDT) decision making which largely relies on the grading. Additionally, these AI‐based algorithms have highlighted the power of being able to detect extremely subtle molecular markers and features that would otherwise be missed by the human eye and could serve as an effective adjunct in diagnostics [5]. Thyroid imaging also has seen AI tools being able to evaluate ultrasonographic findings which include thyroid nodule size, composition, echogenicity as well as vascularity which ultimately help stratify nodules alerting the radiologists as to when further fine‐needle aspiration and cytology should be performed in order to decide on subsequent management for higher risk nodules [4, 7].

When investigating the relationships between socioeconomic deprivation and thyroid cancer diagnosis, literature has been able to show that individuals from a more deprived area socioeconomically had a lower incidence of thyroid cancer diagnosis when compared to patients from a less deprived area [8]. This may reflect a disparity in the ability to access healthcare services but also highlights different health‐seeking behaviours amongst the two groups as well as potential variations in environmental exposure [8]. Molecular testing has been heavily referenced in the literature as a recommendation in cases where traditional cytology results are indeterminate to better inform MDT decision making but should be tailored to individualised patient contexts taking into consideration local availability, costs and patient preferences [9, 10, 11]. Therefore, if addressing socioeconomic factors alongside judicious molecular testing can hope to improve outcomes for patients presenting to clinic, then a potential AI model and its benefits could accentuate surgical pathways.

ChatGPT, a sophisticated language model devised by OpenAI, is one of the most publicly available AI software that employs deep learning methods to generate responses that aim to closely imitate human conversation. It can comprehend and respond to the complexities of human language, making it capable of constructing appropriate and accurate replies to an array of prompts. A thyroid MDT is a group of healthcare professionals that specialise in the diagnosis, treatment and management of thyroid‐related disorders such as thyroid cancer. Some members of this team include ear, nose and throat (ENT) and endocrine surgeons, radiologists, histopathologists, oncologists, cancer specialist nurses and dieticians, amongst others. The growing incidence of thyroid cancer by 65% in the UK over the last decade has meant that these services can often be stretched [12]. This study aims to investigate whether there is a role for AI software such as ChatGPT in providing accurate thyroid MDT outcomes when it comes to suspected thyroid cancer patients. The study hopes to provide some insight into a potential role for AI alongside clinicians in augmenting workflows within healthcare and ultimately providing better patient care.

## 2 Methods

A retrospective study was conducted looking at 30 unique thyroid MDT outcomes for patients discussed at a busy district general hospital in the United Kingdom. Baseline characteristics such as patient gender, age as well as radiological and/or histological features of thyroid disease ranging from benign to malignant lesions were collected. ChatGPT‐4 (OpenAI, San Francisco) was utilised to answer questions posed to the engine to generate management plans for these patients. ChatGPT‐4 was loaded with the most recent British Thyroid Association (BTA) Guidelines for Management of Thyroid Cancer [13]. Our hospital's MDT was treated as the baseline gold standard comparator and when ChatGPT‐4 made multiple recommendations these were taken in the order they were being presented. When ChatGPT‐4's first recommendation matched our MDT's outcome this was regarded as level 1 concordance (Y1). When the second treatment recommendation matched it was level 2 concordance (Y2) etc. all the way up to level 4 concordance; discordant was a further classification where ChatGPT‐4's recommendations did not match our MDT.

## 3 Results

**FIGURE 1:**
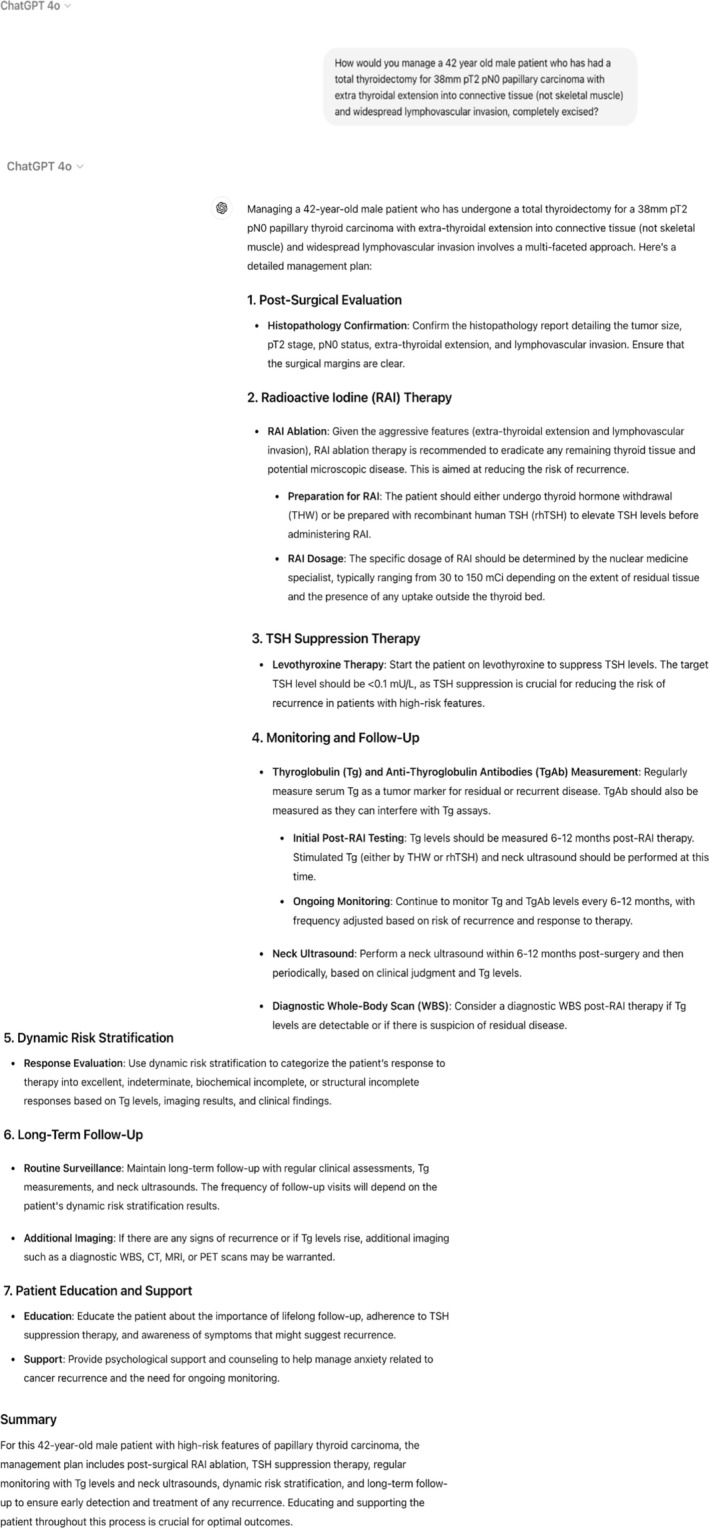
A representative example of a posed clinical scenario to ChatGPT‐4.

Between October 2023 and May 2024, 30 thyroid cases were discussed in our head and neck MDT. They had a mean age of 58 (*n* = 24 female, *n* = 6 male). A representative example of a question for a clinical scenario posed to ChatGPT‐4 is seen below in Figure 1. The MDT's outcome had a 100% concordance with the official guidance from the British Thyroid Association Guidelines for the Management of Thyroid Cancer [13]. When looking at concordance (Table 1) between ChatGPT‐4 and our MDT's decision, the highest level of concordance Y1 was seen in 67% of cases while 13% of cases were completely discordant.

**TABLE 1:** Distribution and concordance rate between MDT and ChatGPT‐4's recommendations for managing suspected thyroid cancer cases.

Level of concordance between MDT and ChatGPT‐4's recommendations	*N* = 30	Percentage concordance %
Y1	20	67
Y2	5	17
Y3	1	3
Y4	0	0
Discordant	4	13
Demographics	Male = 6	Average age = 56.8
	Female = 24	Average age = 58.4
	Pre operative histology *N* = 17	Post operative histology *N* = 13
	Papillary thyroid carcinoma (suspected or confirmed) *N* = 19 Follicular thyroid carcinoma (suspected or confirmed) *N* = 11	
	Neck lymph node involvement *N* = 7	
	Average size of lesion (maximal dimension) = 27.1 mm	

## 4 Discussion

To our knowledge, this is the first study looking at AI's use in thyroid MDTs. In 2021, Watson for Oncology (WFO) based in Cambridge devised an AI tool to assist clinicians in decisions for the management of colorectal cancer management, showing an 81.52% concordance rate between AI and MDT [14]. Kim et al. reported concordance rates as low as 46.4% with a focus on higher risk tumours [15]. Our thyroid MDT outcomes for the 30 scenarios had 100% concordance with the BTA Guidelines for Management of Thyroid Cancer allowing them to be used as a reliable benchmark [13]. In each of the 30 scenarios assessed, ChatGPT‐4 identified the need for complex decision making under the auspices of a thyroid MDT arrangement and followed with suggestions based on the guidelines, accounting for the unique aspects of the case. Additionally, what was very impressive was ChatGPT‐4's ability to accurately take into consideration patient comorbidities as well as age when deciding whether it is advisable to proceed with major surgery.

It was encouraging to see that 67% of the scenarios were the highest level of concordance with the thyroid MDT, with 20% still being Y2 and Y3, which still mentioned the appropriate treatment plan but may have prioritised them lower down on the list of suggestions; thus, 87% of scenarios would have led to the patient having appropriate treatment as per the BTA guidelines for the management of thyroid cancer. This is important to note, as while the study rewards the order of mentioning thyroid MDT outcome plans, the reality is that in clinical practice, the MDT outcome will be tailored to the patient and may encompass options mentioned from Y1 to Y3, for example, in a unifying treatment plan.

In two of the discordant cases, the fine needle aspiration cytology was reported as Thy3a. It was apparent that ChatGPT‐4 struggled with thy3a histology; however, when the queries were repeated with prompts in the questions clarifying Thy3a as ‘atypia’ it accurately identified the correct outcome from the BTA guidelines. This suggests subtle differences in the way AI pools its information from the guidelines and how they can be affected by the semantics within the question. Another discordant scenario was for a 6 cm neck lump in the right level 2 and 3 neck, with ultrasound appearances consistent with lymphoma of the thyroid but core biopsies suggesting primary thyroid carcinoma. Our MDT suggested fine needle aspiration and cytology of the thyroid gland, whilst total thyroidectomy with comprehensive neck dissection was encouraged by ChatGPT‐4. This ultimately did end up being the MDT outcome for this patient on subsequent MDTs, but at the time was not appropriate to proceed straight to major surgery without confirming thyroid histology. This highlights a potential weakness in AI in deciphering complex ‘multiple diagnosis’ scenarios, as well as a gap in BTA for the management of lymphoma. AI will only be able to generate MDT outcomes if it is able to pool this information from reliable evidence‐based guidelines that are inputted. The final discordant scenario involved a right lobe 14.4 mm U5 Thy 5 nodule, which our MDT recommended for right hemithyroidectomy, whilst ChatGPT‐4 suggested total thyroidectomy. Interestingly, similar scenarios came up before and were the highest level of concordance between the two (Y1) implying some level of inter‐scenario variability depending on the way the patient factors are being inputted.

AI has been referenced in the literature for its application in thyroid cytopathology malignancy predictions as well as ultrasound staging with the view of optimising surgical pathways by streamlining and enhancing MDT decision making [4, 5, 6, 7, 11]. Dov et al. were able to design an AI algorithm model that was capable of identifying regions of interest on whole slide images from fine needle aspiration slides [16]. They were able to demonstrate an almost perfect concordance with traditional review methods, which is promising given that it would decrease the time and effort histopathologists spend screening certain cases and allowing them more time to focus on more complex patients. While these results were promising with a relatively larger sample than ours (*N* = 109), the algorithm was trained and tested on a specific dataset from a single hospital; thus, its applicability to diverse populations and different slide preparation techniques is unknown [16]. Wong et al. similarly have shown the innovative ability of AI in automating the analysis of thyroid cytology slides, showing an increased accuracy in the diagnosis as well as classification of thyroid specimens [5]. Overcoming technical barriers of digitising cytological samples will be crucial as these are different from histology specimens due to the variations in staining and cellular dispersion [5]. Fiorentino et al. suggest an increased sensitivity and specificity of thyroid cytopathology using AI models with a focus on diagnostic accuracy of indeterminate thyroid nodules [4]. A clear strength is being able to act as an adjunct in cases where there is diagnostic uncertainty to aid MDT decision making. There is an emphasis on standardisation of cytological diagnoses to lead to more consistent and reproducible results across different hospitals [4]. Fiorentino et al. highlighted the difficulties of safely integrating these AI tools into existing clinical workflows and the need for more user‐friendly interfaces, rigorous clinician training as well as further validation studies in an attempt to complement MDT decision making as opposed to completely replace clinicians [4]. Cao et al. published an extensive review of AI software in thyroid ultrasonography encompassing disease detection, segmentation, differentiation between benign and malignant nodules as well as prediction of metastasis to cervical lymph nodes [7]. In addition to reducing observer variability, AI was shown to improve diagnostic accuracy by automating and standardising image analysis; however, its success will depend on AI models being derived from large, diverse and well‐annotated datasets which are currently scarce [4].

Limiting factors for previous AI software include high costs and difficulties of implementation into clinical practice, but the most recent iterations of large language models such as ChatGPT‐4 are cost‐effective. This processing of data and generation of results takes a fraction of the time taken by humans, with potentially huge time savings if shown to be accurate and reliable. With a pressed national health service and a growing demographic that is thyroid cancer, these MDTs are often oversubscribed, and so having a seamless software that can generate accurate evidence‐based outcomes in the absence of clinicians could free their expertise up to meet the demands of other aspects of the ENT service. However, in light of recent cybersecurity attacks across the National Health Service, coupled with the importance of maintaining patient confidentiality, it is crucial to emphasise and focus on the ethical considerations surrounding the introduction of external software. At present, despite high concordance, there is no role for AI to solely generate outcomes for thyroid MDT cases without being counter‐checked by a clinician and so would end up doubling the workload but would result in fewer clinicians overall needing to present at a singular time slot. This could propose a cost‐effective alternative but could pose a problem as responsibility for outcomes would be solely attributed to this one clinician. A limitation of our study is not having a prospective validation of the AI software in MDT decision making, which should be a focus in future studies to better analyse real‐time benefit. As this is a retrospective study, it is unfortunately subjected to inherent biases on data that has been previously collected as opposed to standardising the study from the beginning and ensuring all patient information and/or the accuracy of electronic records are maintained in order to produce more rigorous data. Where ChatGPT‐4 has relied on inputted guidelines, this poses a potential hurdle as, although BTA is the gold standard, the guidelines can be interpreted differently, and MDT decision‐making has further nuances that AI models may not be able to account for. An important consideration is that in a cancer subtype where guidelines may not be as rigorous as BTA, AI may not perform as well as it did in our study. Another limiting factor is that the MDT radiologist and histopathologist would present their contributions and may addend the reports that would be inputted into the AI software, thus not allowing the program to maximise giving an accurate outcome. The method of subdividing concordance levels is a constraint whereby it assumes AI's order of recommendations is the exact order they should be considered for that individual scenario, whereas in reality, it will be a combination of all the suggestions that are acceptable as per BTA guidelines; hence, differentiating between acceptable and completely incorrect becomes difficult with concordance levels. Also, the fact that they have been collected by one clinician is a source of subjectivity in interpreting the AI‐generated outcomes. Additionally, this study had a small sample size, solely analysed thyroid MDTs, and focused on the use of one AI model, meaning our results may not be representative for other AI models and ENT malignancies. Ideally, this study would be repeated at multiple centres to gauge whether these results could be replicated, where perhaps expertise may change the MDT outcome for complex scenarios. In addition, the variability in ultrasound reporting at our trust was problematic and could have led to ChatGPT‐4 not being able to accurately provide a treatment option.

## 5 Conclusion

AI has the potential to optimise clinical medicine, in particular with respect to therapeutics surrounding MDT decision making. It is cheap, easy to use and, if done correctly, can be a safe way to provide evidence‐based treatment plans for the management of thyroid cancer, freeing some clinicians from this role and allowing them to meet other demands of the ENT service. There is a need for more prospective, multi‐centre, larger studies to be conducted to further replicate our concordance rates, as well as look at other subsets of head and neck cancers. Outcomes should still be counterchecked by a clinician; therefore, doubling work as well as focusing the responsibility on that single clinician. The ability of ChatGPT‐4 to understand complex patient scenarios and formulate an evidence‐based treatment plan in line with approved guidelines is exciting for the future and offers an opportunity to explore AI in other facets of medicine.

## Author Contributions

Amir Habeeb designed the work, acquired and analysed data, drafted, revised and approved the manuscript. Amir Habeeb agrees to be accountable for all aspects of the work. Kim Hui Lim acquired and analysed data and drafted the manuscript. Samuel Chan designed the work, acquired and analysed data, revised and approved the manuscript. Xenofon Kochilas revised and approved the manuscript. Nazir Bhat revised and approved the manuscript. Furrat Amen revised and approved the manuscript.

## Ethics Statement

This publication has complied with ethical standards. All data were collected through a hospital audit, were anonymised and disposed of in line with data protection guidance.

## Conflicts of Interest

The authors declare no conflicts of interest.

### Peer Review

The peer review history for this article is available at https://www.webofscience.com/api/gateway/wos/peer‐review/10.1111/coa.14305.

## Data Availability

Data sharing can be made available for this manuscript on request from the authors.

## References

[1] J. He , S. L. Baxter , J. Xu , J. Xu , X. Zhou , and K. Zhang , “The Practical Implementation of Artificial Intelligence Technologies in Medicine,” Nature Medicine 25, no. 1 (2019): 30–36, https://pubmed.ncbi.nlm.nih.gov/30617336/. 10.1038/s41591-018-0307-0 PMC6995276 30617336

[2] K. W. Johnson , J. Torres Soto , B. S. Glicksberg , et al., “Artificial Intelligence in Cardiology,” Journal of the American College of Cardiology 71, no. 23 (2018): 2668–2679, https://pubmed.ncbi.nlm.nih.gov/29880128/. 29880128 10.1016/j.jacc.2018.03.521

[3] F. Jiang , Y. Jiang , H. Zhi , et al., “Artificial Intelligence in Healthcare: Past, Present and Future,” Stroke and Vascular Neurology 2, no. 4 (2017): 230–243, https://pubmed.ncbi.nlm.nih.gov/29507784/. 29507784 10.1136/svn-2017-000101 PMC5829945

[4] V. Fiorentino , C. Pizzimenti , M. Franchina , et al., “The Minefield of Indeterminate Thyroid Nodules: Could Artificial Intelligence Be a Suitable Diagnostic Tool?,” Diagnostic Histopathology 29, no. 8 (2023): 396–401.

[5] C. M. Wong , B. E. Kezlarian , and O. Lin , “Current Status of Machine Learning in Thyroid Cytopathology,” Journal of Pathology Informatics 14 (2023): 100309. 37077698 10.1016/j.jpi.2023.100309 PMC10106504

[6] K. Chain , T. Legesse , J. E. Heath , and P. N. Staats , “Digital Image‐Assisted Quantitative Nuclear Analysis Improves Diagnostic Accuracy of Thyroid Fine‐Needle Aspiration Cytology,” Cancer Cytopathology 127, no. 8 (2019): 501–513, https://onlinelibrary.wiley.com/doi/full/10.1002/cncy.22120. 31150162 10.1002/cncy.22120

[7] C. L. Cao , Q. L. Li , J. Tong , et al., “Artificial Intelligence in Thyroid Ultrasound,” Frontiers in Oncology 13 (2023), https://pubmed.ncbi.nlm.nih.gov/37251934/. 10.3389/fonc.2023.1060702 PMC10213248 37251934

[8] C. M. Kitahara and A. B. Schneider , “Epidemiology of Thyroid Cancer,” Cancer Epidemiology, Biomarkers & Prevention 31, no. 7 (2022): 1284–1297, https://pubmed.ncbi.nlm.nih.gov/35775227/. 10.1158/1055-9965.EPI-21-1440 PMC9473679 35775227

[9] C. A. Lebbink , T. P. Links , A. Czarniecka , et al., “European Thyroid Association Guidelines for the Management of Pediatric Thyroid Nodules and Differentiated Thyroid Carcinoma,” European Thyroid Journal 11, no. 6 (2022), https://pubmed.ncbi.nlm.nih.gov/36228315/. 10.1530/ETJ-22-0146 PMC9716393 36228315

[10] M. Dell'Aquila , C. Gravina , A. Cocomazzi , et al., “A Large Series of Hyalinizing Trabecular Tumors: Cytomorphology and Ancillary Techniques on Fine Needle Aspiration,” Cancer Cytopathology 127, no. 6 (2019): 390–398, https://pubmed.ncbi.nlm.nih.gov/31135104/. 31135104 10.1002/cncy.22139

[11] V. Fiorentino , M. Dell' Aquila , T. Musarra , et al., “The Role of Cytology in the Diagnosis of Subcentimeter Thyroid Lesions,” Diagnostics (Basel) 11, no. 6 (2021), https://pubmed.ncbi.nlm.nih.gov/34204172/. 10.3390/diagnostics11061043 PMC8230300 34204172

[12] Thyroid cancer statistics , “Cancer Research UK [Internet],” (2024), https://www.cancerresearchuk.org/health‐professional/cancer‐statistics/statistics‐by‐cancer‐type/thyroid‐cancer.

[13] P. Perros , K. Boelaert , S. Colley , et al., “Guidelines for the Management of Thyroid Cancer,” Clinical Endocrinology 81 (2014): 1–122, 10.1111/cen.12515. 24989897

[14] Z. Jie , Z. Zhiying , and L. Li , “A Meta‐Analysis of Watson for Oncology in Clinical Application,” Scientific Reports 11, no. 1 (2021), https://pubmed.ncbi.nlm.nih.gov/33707577/. 10.1038/s41598-021-84973-5 PMC7952578 33707577

[15] E. J. Kim , H. S. Woo , J. H. Cho , et al., “Early Experience With Watson for Oncology in Korean Patients With Colorectal Cancer,” PLoS One 14, no. 3 (2019), https://pubmed.ncbi.nlm.nih.gov/30908530/. 10.1371/journal.pone.0213640 PMC6433269 30908530

[16] D. Dov , S. Z. Kovalsky , Q. Feng , et al., “Use of Machine Learning–Based Software for the Screening of Thyroid Cytopathology Whole Slide Images,” Archives of Pathology & Laboratory Medicine 146, no. 7 (2022): 872–878. 34669924 10.5858/arpa.2020-0712-OA

